# Beyond Social Structures: Using MIDUS To Understand Psychological Processes and Social Inequalities

**DOI:** 10.1093/geroni/igaf122.1539

**Published:** 2025-12-31

**Authors:** Deborah Carr

**Affiliations:** Boston University, Boston, Massachusetts, United States

## Abstract

MIDUS is a large multi-wave multicohort longitudinal study of midlife and aging in the United States. The large sample enables critical explorations of intersectionality, or the ways that gender, race, age, disability status, socioeconomic status, obesity, and other social identities intersect to shape key outcomes spanning physical, emotional, and cognitive health. MIDUS is distinct from other U.S.-based and international studies of aging because it collects extensive and novel data on psychosocial processes that link social and structural factors to individual-level outcomes. This presentation will focus on one subset of these questions: perceived discrimination. Perceptions of both structural/institutional discrimination, like blocked opportunities in hiring and workplace advancement, and interpersonal discrimination, which encompasses microaggressions like being treated condescendingly, are key mechanisms linking structural positions to health and well-being. This presentation will feature key publications from the MIDUS and new empirical analyses that demonstrate the value of understanding institutional and interpersonal discrimination, and how these experiences are shaped by social contexts. For example, results from new analyses reveal: (a) how the impacts of disability status on workplace discrimination vary based on workplace requirements as defined by O*NET data, and (b) the complex ways that disability status and obesity intersect to shape experiences of interpersonal and institutional discrimination. These studies build on prior work demonstrating how workplace discrimination mediates the association between disability status and psychological well-being over the life course. Study results can inform policies and practices to enhance population health.

